# Case report: Central-pituitary hypothyroidism concurrent with hyperadrenocorticism without pituitary macroadenoma in a Miniature Schnauzer dog

**DOI:** 10.3389/fvets.2023.1257624

**Published:** 2023-09-25

**Authors:** Yeon Chae, Taesik Yun, Yoonhoi Koo, Dohee Lee, Mhan-Pyo Yang, Hakhyun Kim, Byeong-Teck Kang

**Affiliations:** Laboratory of Veterinary Internal Medicine, College of Veterinary Medicine, Chungbuk National University, Cheongju, Republic of Korea

**Keywords:** central-pituitary hypothyroidism, hyperadrenocorticism, Miniature Schnauzer dog, multiple endocrinopathies, secondary hypothyroidism

## Abstract

Multiple endocrine disorders are uncommon in veterinary medicine, and the disease combination is usually related to hypercortisolism or autoimmunity. Central-pituitary hypothyroidism, also refer to secondary hypothyroidism, can be caused by hypercortisolemic conditions and is well-recognized in human medicine. However, central hypothyroidism, including pituitary hypothyroidism, concurrent with hyperadrenocorticism, is rarely reported in veterinary medicine. A 7-year-old, intact female Miniature Schnauzer presented with generalized alopecia, scale, and pruritus and was diagnosed with superficial pyoderma and *Malassezia* dermatitis. Hormonal tests were performed, and the results indicated multiple endocrinopathies with a combination of non-adrenal dependent hyperadrenocorticism and central-pituitary hypothyroidism. Magnetic resonance imaging (7 T) and high-resolution research tomography positron emission tomography were performed to differentiate neuroendocrine tumors; however, no lesion was found in the hypothalamic to pituitary region. Hyperadrenocorticism was managed first to control endocrinopathy. After controlling hypercortisolism, a weak elevation of free thyroxine (T4) was revealed, whereas total T4 and thyroid-stimulating hormone (TSH) were still undetectable, and hypothyroidism management was added. About 9 months after the management, both endocrine diseases were well controlled, and clinical signs improved; however, serum TSH was unmeasured consistently. This case study describes a case of multiple endocrinopathies in a Miniature Schnauzer dog diagnosed with central-pituitary hypothyroidism concurrent with non-adrenal dependent hyperadrenocorticism without pituitary macroadenoma.

## 1. Introduction

Multiple endocrine disorders, including more than one endocrine disorder, have been reported uncommonly and are not well-established in veterinary medicine. The prevalence of multiple endocrine disorders in dogs was low, representing 0.3% of canine patients and 2.3% of the total canine patients with endocrinopathy (1). A previous study reported that combination of disorders included diabetes mellitus and hyperadrenocorticism 57.1%, hypoadrenocorticism and hypothyroidism 22.9%, diabetes mellitus and hypothyroidism 28.6% in the dogs with multiple endocrinopathies (1). Another study reported that the dogs with diabetes mellitus and hyperadrenocorticism were 6.65%, diabetes mellitus and hypothyroidism were 0.6%, and hyperadrenocorticism and primary hypothyroidism were 0.53% among all dogs with endocrine disease (2). Canine hypothyroidism is a common canine endocrinopathy; however, central hypothyroidism has been rarely documented and is less than 5% of all cases of canine hypothyroidism (3, 4). Central-pituitary hypothyroidism, also refer to secondary hypothyroidism, results from a loss of pituitary thyroid-stimulating hormone (TSH) secretion and is usually associated with an expanding pituitary tumor, pituitary malformation, and pituitary destruction due to radiation therapy or surgery (3, 5). Additionally, a possible breed predisposing to central hypothyroidism in Miniature Schnauzer has been reported previously (6). In human medicine, central-pituitary hypothyroidism is a potential, uncommon, and reversible condition due to hypercortisolemic conditions; however, it is controversial in veterinary medicine (7–9). In veterinary medicine, central hypothyroidism concurrent with hyperadrenocorticism was rarely reported in two cases accompanied by pituitary macroadenoma (10, 11). This case report describes a case of multiple endocrinopathies concurrent with non-adrenal dependent hyperadrenocorticism and central-pituitary hypothyroidism without pituitary macroadenoma in Miniature Schnauzer.

## 2. Case presentation

**Figure 1:**
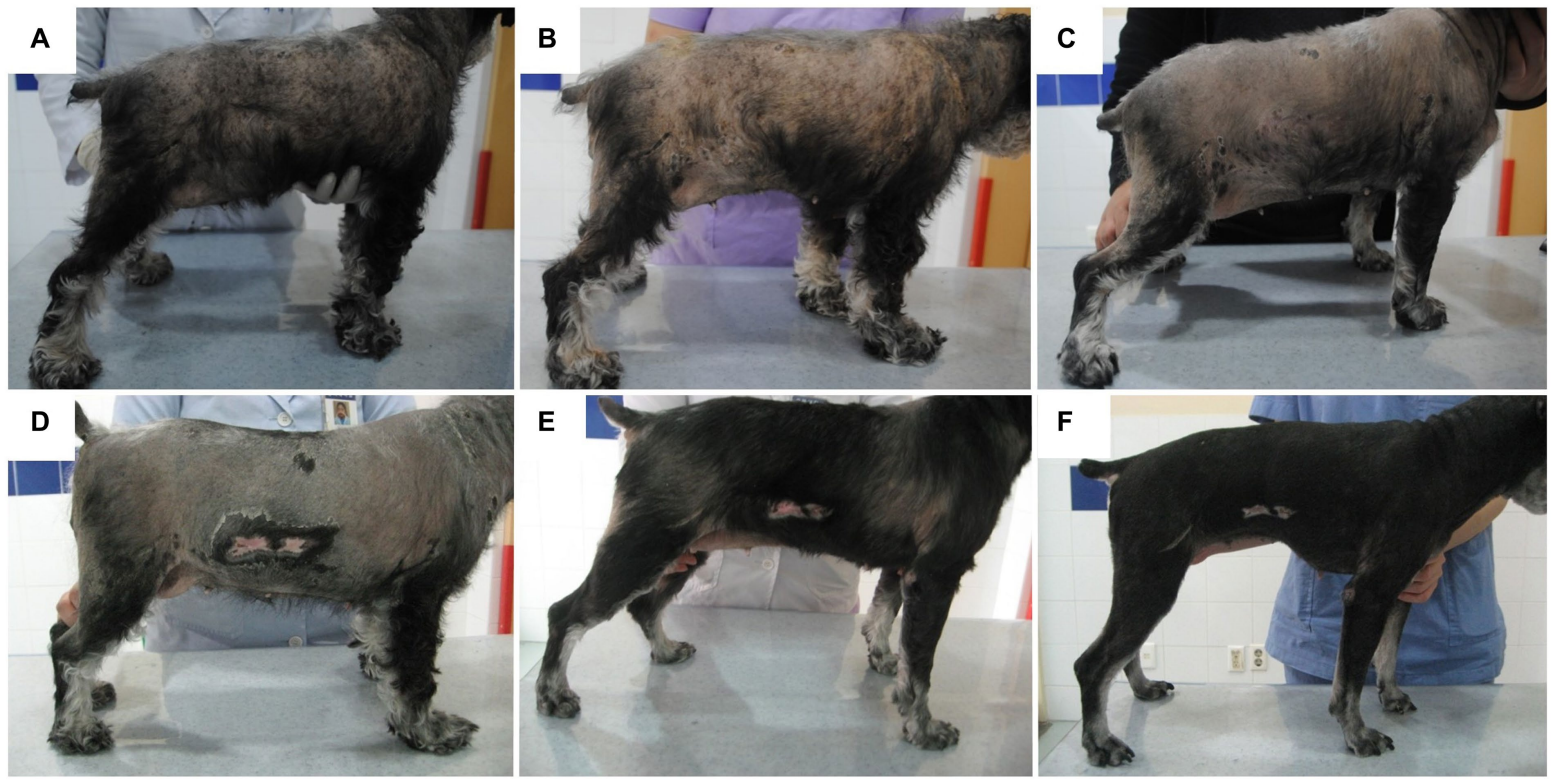
Schematic diagram photography of cutaneous lesion improvement. Generalized truncal alopecia and scale with hyperpigmentation were presented before the treatment **(A)**. No significant improvement was observed 2 weeks after the management of superficial pyoderma and Malassezia dermatitis **(B)**. Mild improvement of papule and scale was identified 1 month after hyperadrenocorticism management **(C)**. Generalized skin lesions, including alopecia, were improved after additional hypothyroidism management at 1 month **(D)**, 3 months **(E)**, and 5 months **(F)**.

**Figure 2:**
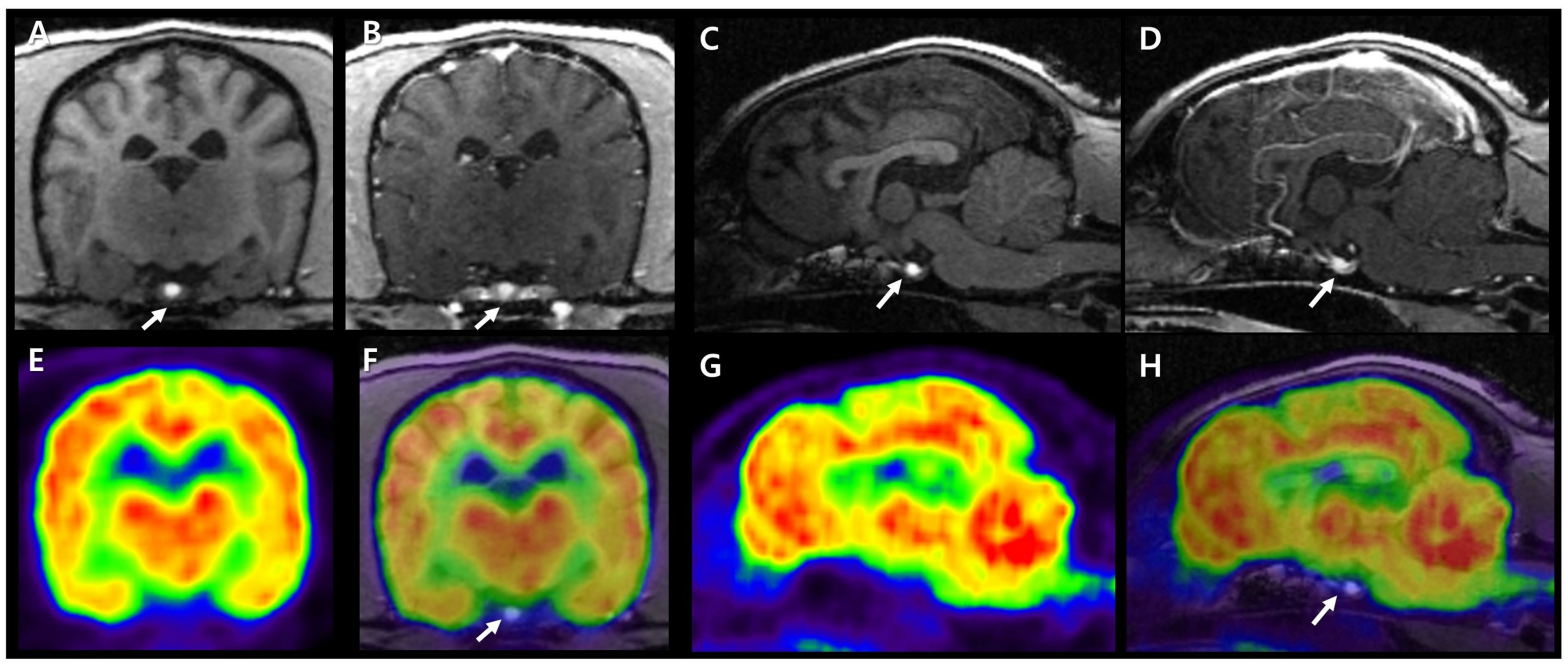
7 T MRI and FDG-PET characteristics of a canine pituitary region. Pre- and post-contrast T1 images in transverse **(A,B)** and sagittal plane **(C,D)**. Neurohypophysis is presented as a hyperintense signal in the center of the pituitary gland (arrows). The adenohypophysis is surrounding neurohypophysis with an isointense signal in T1 image and is enhanced after gadolinium injection. FDG-PET images in transverse and sagittal planes **(E,G)**. Fusion of T1 MRI and FDG-PET images in transverse and sagittal planes **(F,H)**. On PET images, reddish to yellowish color represents high FDG uptake and bluish to greenish color represents low FDG uptake. The pituitary gland showed lower glucose metabolism than cerebral cortex and white matter. MRI, magnetic resonance imaging; FDG-PET, fluorodeoxyglucose-positron emission tomography.

A 7-year-old, intact female Miniature Schnauzer weighing 7.4 kg was admitted because of generalized alopecia, scale, and pruritus. Physical examination revealed bilateral symmetric truncal alopecia with hyperpigmentation in the dorsum, abdomen, axilla, and inguinal region (Figure 1A). Infectious dermatitis, which is associated with a mixed infection of bacteria and *Malassezia,* was identified on cytology and culture. In the hematologic examination, normocytic, normochromic, nonregenerative anemia (packed cell volume, 35.6%; reference interval [RI], 37.3–61.7%) was identified in complete blood count, and hypercholesterolemia (total cholesterol, 967 mg/dL; RI, 135–270 mg/dL), hypertriglyceridemia (triglyceride, 470 mg/dL; RI, 21–116 mg/dL), and increased activity of liver enzymes (alkaline phosphate, 316 IU/L; RI, 29–97 IU/L) were revealed from serum chemistry analysis. There were no abnormal findings on thoracic radiography, but hepatomegaly and urinary bladder calculi were identified on abdominal radiography. Abdominal ultrasonography revealed a hyperechoic liver and gall bladder mucocele. The size of both adrenal glands was normal (left cranial pole, 5.5 mm; right cranial pole, 4.9 mm), and the morphologic abnormalities were not evident. Secondary infection due to concurrent endocrine disease was suspected, and hormonal tests were performed. An exogenous adrenocorticotrophic hormone (ACTH; Synacthen, Novartis Pharm., Basel, Switzerland) stimulation test was performed and revealed high post-ACTH cortisol (1,156 nmol/L, 41.9 μg/dL) that exceeded 600 nmol/L and was approximately six times greater than pre-ACTH cortisol (183 nmol/L, 6.65 μg/dL). A low-dose dexamethasone suppression (LDDS) test was performed to distinguish between pituitary and adrenal disease. After administration of dexamethasone (Je-il Pharm., Daegu, Korea), the concentration of 4-h post-dexamethasone serum cortisol (<28 nmol/L) was suppressed by less than 50% of baseline concentration (129 nmol/L) and 6-h post-dexamethasone serum cortisol measured as 32 nmol/L. Therefore, adrenal-dependent hyperadrenocorticism was excluded, and pituitary-dependent hyperadrenocorticism (PDH) was suspected. Additional hormonal tests were performed because PDH can be associated with other pituitary-dependent endocrinopathies. Hormonal assessments of the hypothalamic–pituitary-thyroid (HPT) axis were performed and revealed low serum total thyroxine (T4) (<0.3 μg/dL; RI, 1.0–4.0 μg/dL), low free T4 (<0.3 ng/dL; RI, 0.6–3.7 ng/dL), and low serum levels of TSH (<0.03 ng/mL; RI, 0.05–0.42 ng/mL). Plasma TSH concentration after stimulation with thyrotropin-releasing hormone (TRH; Cerebrain, Shinpoong Pharm., Seoul, Korea) also revealed low levels at 10, 20, 30 min, and 4 h (<0.03 ng/mL, respectively). Total T4 and free T4 concentrations were also undetectable before and 4 h after stimulation with TRH (total T4, <0.3 μg/dL; free T4, <0.3 ng/dL, respectively). Based on these results, a pituitary endocrine tumor was suspected, and intracranial imaging was performed using 7 T magnetic resonance imaging (MRI) and high-resolution research tomography positron emission tomography (HRRT-PET) under general anesthesia. The dog was fasted for 12 h, and 18F-fluorodeoxyglucose (FDG) was administered at 0.14 mg/kg intravenously. Sixty min after the FDG injection, the FDG-PET scan was conducted for 30 min on the HRRT device (ECAT HRRT; Siemens, Knoxville, TN, United States). Immediately after the FDG-PET scan, pre- and post-contrast T1- weighted images of the brain were obtained using a 7 T-MRI scanner (Magnetom 7 T; Siemens, Berlin, Germany; Figures 2A–D). In the transverse pre-contrast T1-weighted image, a hyperintense region, indicating neurohypophysis, was present in the center of the pituitary gland. The size of the pituitary gland was measured (height, 3.7 mm, RI: 3.0–7.5 mm; width, 4.8 mm, RI: 4.0–9.0 mm) on the transverse post-contrast T1 weighted image and the pituitary height/brain area (P/B) ratio was 0.2 (RI, ≤0.31). The standardized uptake value of the pituitary gland (mean, 0.32; max, 0.47) was lower than those of the gray matter (mean, 0.96; max, 1.02) and caudal colliculus (mean, 1.69; max, 2.44; Figures 2E–H). No evidence of pituitary macroadenoma or abnormal findings was observed in the brain. As pituitary microadenoma cannot be ruled out, insulin-like growth factor (IGF-1) was measured to differentiate growth hormone-secreting abnormalities. It was measured by a reference laboratory (Diagnostic Center for Population & Animal Health, Michigan State University, Lansing, MI), and the result (7 nmol/L; RI, 4–95 nmol/L) was indicated as normal. Therefore, we concluded that the patient had multiple endocrinopathies, including non-adrenal dependent hyperadrenocorticism and central-pituitary hypothyroidism, without a pituitary macroadenoma.

To treat infectious dermatitis, amoxicillin/clavulanic acid (25 mg/kg PO q12h), itraconazole (5 mg/kg PO q24h), and benzoyl peroxide shampoo were prescribed for 2 weeks; however, no significant improvement was observed (Figure 1B). To control the endocrinopathy and infectious dermatitis, which can be caused by hyperadrenocorticism or hypothyroidism, the medication for hyperadrenocorticism, trilostane (1 mg/kg PO q12h), was prescribed first because hypercortisolism can suppress the HPT axis. After the control of the hypercortisolism (post-ACTH stimulation test results 5.12 ug/dL), total T4 and TSH concentrations were still undetectable, whereas free T4 (0.435 ng/dL) revealed weak elevation (Table 1). L-thyroxine (0.02 mg/kg PO q12h) was administered after the stabilization of hyperadrenocorticism to control hypothyroidism and to pursue rapid clinical improvement. Some responses, such as improvement of papule and scale, were identified after the control of hyperadrenocorticism and infectious dermatitis (Figure 1C), whereas generalized alopecia was improved after levothyroxine administration (Figures 1D–F). After 6 months of levothyroxine administration (7 months of hyperadrenocorticism management), increased total T4 (2.80 ug/dL) and free T4 (5.78 ng/dL) were identified with a general improvement in body condition (Figure 1F), whereas serum TSH was still undetectable. About 9 months after the control of the hyperadrenocorticism, the serum cortisol concentration of pre- and post-ACTH stimulation test was revealed, 2.55 ug/dL and 2.89 ug/dL, respectively. At the same time point, total T4 was 2.24 ug/dL, and free T4 was 5.24 ng/dL, whereas TSH was unmeasured consistently (Table 1).

**Table 1:** Monitoring management for a dog with non-adrenal dependent hyperadrenocorticism and central-pituitary hypothyroidism.

Parameters			Initial presentation	Following after management						
ACTH stimulation test	0 h cortisol (μg/dL)		6.65 (RI: 0.5–4)	4.06	3.74	NA	7.23	2.13	NA	2.55
	1 h cortisol (Therapeutic target, 1–5; μg/dL)		41.9 (RI: 8–20)	12.4	5.12	NA	11.3	2.48	NA	2.89
Thyroid hormonal assessment	Total T4 (Therapeutic target, 3–4; μg/dL)		<0.3 (RI: 1.0–4.0)	NA	<0.3	3.8	NA	NA	2.80	2.24
	Free T4 (ng/dL)		<0.3 (RI: 0.6–3.7)	NA	0.435	>6.0	NA	NA	5.78	5.24
	TSH (ng/mL)		<0.03 (RI: 0.05–0.42)	NA	<0.03	<0.03	NA	NA	<0.03	<0.03
Treatment	Trilostane	Dose	–	1 mg/kg q12h	2 mg/kg q12h	2 mg/kg q12h	2 mg/kg q12h	4 mg/kg q12h	4 mg/kg q12h	4 mg/kg q12h
		Prescribed duration	–	1 week	1 month	2 months	5 months	6 months	7 months	10 months
	L-T4	Dose				0.02 mg/kg q12h	0.02 mg/kg q12h	0.02 mg/kg q12h	0.02 mg/kg q12h	0.02 mg/kg q12h
		Prescribed duration				1 month	4 months	5 months	6 months	9 months

ACTH, adrenocorticotrophic hormone; L-T4, L-thyroxine; NA, not available; RI, reference interval.

## 3. Discussion

Multiple endocrinopathies are uncommon in veterinary medicine, and the most common combination of endocrine disorders is hyperadrenocorticism and diabetes mellitus (1, 2). The etiology of these two diseases is usually unrelated; however, insulin resistance secondary to hyperadrenocorticism may influence diabetes mellitus occurrence (1, 2). The next most common multiple endocrinopathy is hypothyroidism with hypoadrenocorticism or diabetes mellitus, which can have a potential association with autoimmune poly-endocrinopathy (1, 2). In this case, the Miniature Schnauzer dog, which has breed predisposing hypothyroidism, was diagnosed with non-adrenal dependent hyperadrenocorticism and central-pituitary hypothyroidism without pituitary macroadenoma.

Because hyperadrenocorticism dogs have clinical signs similar to those of hypothyroid dogs and may have low total T4 concentrations, the differential diagnosis between hypothyroidism and euthyroid sick syndrome can be challenging. Moreover, dogs with primary hypothyroidism have low total T4 levels and high TSH concentrations (3). In dogs with non-thyroidal-illness syndrome, including hyperadrenocorticism, the occurrence of high TSH concentration with low T4 is uncommon (9, 12–14). The suppression of the HPT axis caused by hypercortisolism is well established in humans (7). In veterinary medicine, the effects of exogenous and endogenous glucocorticoids on the HPT axis have also been investigated in dogs (9, 14–19). However, central-pituitary hypothyroidism due to hyperadrenocorticism is diagnosed uncommonly, although it can be theoretically common. A previous case study reported a dog with central-pituitary hypothyroidism and PDH caused by pituitary macroadenoma (11). Differently, in our case, the dog with non-adrenal dependent hyperadrenocorticism and central-pituitary hypothyroidism was diagnosed by hormonal assessment and showed no evidence of abnormal lesions in the brain on 7 T MRI and HRRT-PET. However, even if no lesions are identified through brain MRI, microadenoma cannot be ruled out. In human medicine, microadenomas are more common than macroadenomas, and Cushing’s disease is often caused by microadenomas (7, 20, 21). Furthermore, a higher prevalence of central hypothyroidism is reported in ACTH-secreting microadenomas than in other microadenoma types, which indicates the role of hypercortisolism in the genesis of central hypothyroidism (8). At the hypothalamic level, chronic hypercortisolism can reduce TRH gene expression in the hypothalamic paraventricular nucleus and increase somatostatin release, which in turn inhibits TSH release (7, 22–25). At the pituitary level, hypercortisolism directly inhibits TSH secretion (7, 26). However, even if TSH isoforms are qualitatively defective and have impaired biological activity, they can be quantitatively normal or even elevated and maintain their immunoreactivity in the diagnostic test (27, 28). Therefore, differentiating central-pituitary hypothyroidism requires the TRH stimulation test; however, it is often considered underdiagnosed in dogs due to the low sensitivity of TSH measurement, which cannot detect all isoforms (28). In this case, the dog was diagnosed with central-pituitary hypothyroidism rather than non-thyroidal illness syndrome due to hypercortisolism because TSH and T4 were undetectable before and after TRH stimulation. However, it could not be concluded as the pituitary hypothyroidism secondary to PDH, because hypersecreting endogenous ACTH was not confirmed in this dog. Moreover, the reduction of TSH levels during uncontrolled hypercortisolism can return to normal thyroid function after the cure of PDH, which has been reported in both humans and dogs (7, 14, 29). Although, in this case, post-ACTH stimulation cortisol levels were maintained appropriately after hyperadrenocorticism management, TSH concentration was undetectable until 9 months after treatment. Serum TSH may not have had enough time to recover or may be influenced by levothyroxine medication which can suppress serum TSH concentration. However, suppression of serum TSH concentration which caused by levothyroxine application was identified in euthyroid or primary hypothyroid dogs (5, 30–33), but was not investigated in dogs with central-pituitary hypothyroidism. Therefore, further investigation of sustained TSH suppression and other causes of pituitary hypothyroidism should also be considered in our case.

Another possibility in the current case is a breed-specific predisposition. A previous study reported central hypothyroidism in Miniature Schnauzer (6). These dogs were diagnosed by hormonal assays, including a 3-day-TSH-stimulation test or TRH stimulation test and thyroid scintigraphy, and had no evidence of pituitary and hypothalamic abnormalities in CT scans (6). The genetic background of this breed was suspicious, whereas no disease-causing mutations were found in the TSH-beta gene and the exons of the TRH receptor gene (6). However, TSH release can be decreased in primary hypothyroidism because of TRH receptor desensitization, which is caused by persistent stimulation of thyrotropes *via* the negative feedback loop (30). In laboratory-induced primary hypothyroidism, TSH increases after thyroidectomy and decreases gradually after 3 years; however, it can be detectable and measured within the reference range (30). In this case, loss of TSH-secreting function was identified through the TRH stimulation test. However, differentiating thyroid atrophy caused by decreased TSH secretion from pituitary thyrotrope desensitization due to primary hypothyroidism is challenging because primary hypothyroidism was not discriminated through the TSH stimulation test. Although weak elevation of free T4 was identified after PDH management, total T4 and TSH were still undetectable. Furthermore, the pituitary gland can usually be enlarged secondary to primary hypothyroidism (30). However, in this case, no evidence of an enlarged pituitary gland in the 7 T-MRI scan and no increased avidity of FDG on the HRRT-PET scan were detected, which can help in a more accurate diagnosis of the pituitary lesion (34–37). Based on these findings, central-pituitary hypothyroidism associated with breed-specificity is highly suggested rather than primary hypothyroidism.

Another possible cause of hypercortisolism is adrenal TRH stimulation. TRH is transported to the pituitary *via* the hypothalamic–hypophyseal portal system, which consists of the primary capillary plexus penetrating the median eminence, and directly stimulating TRH receptors on melanotropes in the pars intermedia of the pituitary gland. It releases stored proopiomelanocortin, which is then converted into ACTH and other peptides by prohormone convertases (38). A previous study reported the presence of TRH receptors in the pituitary and adrenal glands of dogs (39). However, plasma cortisol concentration did increase significantly after TRH stimulation, whereas plasma ACTH concentration did not rise significantly (39). This suggests the possibility of hypercortisolism, which can be induced by direct TRH stimulation of the adrenal gland. In this case, hyperadrenocorticism was confirmed by the ACTH stimulation test, and adrenal-dependent hyperadrenocorticism was excluded by the LDDS test. This dog had no evidence of ectopic ACTH secretion such as intrathoracic tumors or neuroendocrine malignancies on radiography and abdominal ultrasonography. No brain lesions, including those in the pituitary to hypothalamic area, were identified on HRRT-PET and 7 T MRI. While it is plausible to speculate that the hypercortisolism identified in this case could have resulted from TRH elevation, which can be increased due to central-pituitary hypothyroidism, in practice, we were unable to measure the endogenous TRH concentration as the TRH assay is not validated in dogs and unmeasured endogenous ACTH because of laboratory limitation.

In human medicine, hypothyroidism causes elevated cortisol levels, presumably due to decreased clearance and blunted negative cortisol feedback in the hypothalamus-pituitary–adrenal axis (40). Furthermore, reduced serum cortisol levels after 5–7 months of levothyroxine therapy in patients with primary hypothyroidism have been reported (41). In veterinary medicine, hypercortisolemic status tend to be controlled prior to conclusive diagnosis or management of hypothyroidism, because of its HPT axis suppressing effect. Also, in this case, hyperadrenocorticism was managed first, but clinical improvement was not clearly identified. After managing concurrent hypothyroidism during treatment for hyperadrenocorticism, the post-ACTH cortisol level and clinical signs showed stabilization at 5 months. This suggests that the management of hypothyroidism influences the control of hyperadrenocorticism. Therefore, in this case, the additional consideration, that secondarily caused elevation of cortisol concentration which is influenced by breed related central hypothyroidism rather than a PDH-HAC from the beginning, should be suspected such as below. The cortisol level that was elevated initially (baseline and post-ACTH) could have been influenced by the generalized infectious dermatitis. Basal and post-ACTH cortisol concentration were reported already within the RI 1 week after trilostane, which usually takes slightly more time to act. At that time, the dog was already on antibiotics for 2 weeks. Additionally, clinical signs and laboratory results for this dog were generally inconsistent with HAC. This dog also seemed to improve significantly only when levothyroxine was added and not with trilostane only. Therefore, to control both concomitant endocrinopathies well, a definitive differential diagnosis between primary and central hypothyroidism is needed because central hypothyroidism can be misdiagnosed due to the effect of hypercortisolism in suppressing HPT axis and hypothyroidism can be speculated to increase the cortisol level.

Theoretically, hypercortisolism concurrent with primary or secondary hypothyroidism that have elevated endogenous TRH can be common in veterinary medicine. However, in authors knowledge, the concept of hyperadrenocorticism secondary to hypothyroidism, which is the state of oversecretion of TRH is not mentioned before in dogs. We think that it might be underdiagnosed these connected diseases because of the limitation of diagnosing methods, such as low sensitivity and specificity of TSH measuring, limited use of TSH and TRH stimulation tests, non-measurement of endogenous ACTH, in veterinary field. Clinicians can face the state of concurrent these two endocrinopathies more commonly than in the literature, but definitive diagnosis still can be challenged because diagnostic methods have limitations as we mentioned above. Therefore, further exploration on the new insight of comorbidity for hyperadrenocorticism and hypothyroidism should be needed.

## Data availability statement

The original contributions presented in the study are included in the article/supplementary material, further inquiries can be directed to the corresponding author.

## Ethics statement

Ethical approval was not required for the studies involving animals in accordance with the local legislation and institutional requirements because this case study was descripted retrospectively from the data obtained for clinical purposes. All the procedures being performed were part of the routine care. Informed consent for all datas in our manuscript were obtained from the owners. Written informed consent was obtained from the participant/patient(s) for the publication of this case report.

## Author contributions

YC: Writing – original draft. TY: Writing – review & editing. YK: Writing – review & editing. DL: Writing – review & editing. M-PY: Writing – review & editing. HK: Writing – review & editing. B-TK: Writing – review & editing.
